# A Pilot Study to Determine the Consistency of Simultaneous Sleep Actigraphy Measurements Comparing All Four Limbs of Patients with Parkinson Disease

**DOI:** 10.3390/geriatrics3010001

**Published:** 2017-12-27

**Authors:** Vineet Prasad, Cary A. Brown

**Affiliations:** 1 Faculty of Rehabilitation Medicine, University of Alberta, Edmonton, AB T6G 2G4, Canada; 2 Department of Occupational Therapy, Faculty of Rehabilitation Medicine, University of Alberta, Edmonton, AB T6G 2G4, Canada; cary1@ualberta.ca

**Keywords:** actigraph, actigraphy, Parkinson disease, sleep, insomnia

## Abstract

Wrist actigraphy is a form of objective sleep measurement that has gained a central role in sleep research and clinical settings. Guidelines for actigraphy recommend placing the monitor on the non-dominant wrist, however, this potentially will be the most involved limb for someone with Parkinson disease, and so alternative placement would be preferred. To-date, there is little published about sleep actigraphy use in Parkinson disease (PD). This study examines the degree of sleep actigraphy score variation in persons with PD when monitors are placed simultaneously on all four limbs. In this study, four participants wore a sleep actigraph on each limb for seven nights. Data from the four actigraphs was compared within each participant to determine the degree of consistency. We found that all of the participants’ sleep efficiency and total sleep time scores were higher in the lower limb than upper limb. There was no notable difference in sleep variables between the dominant arm and non-dominant arm. We concluded that simultaneous actigraphy measurement did not notably vary between dominant and non-dominant arms. However, a discrepancy was seen between upper limbs and lower limbs actigraph scores. Further study is warranted to develop guidelines for sleep actigraphy use in this population.

## 1. Introduction

In recent years, there has been a growing volume of research literature that is related to the use of sleep actigraphy. Actigraphy has gained a central role as an objective assessment tool in sleep medicine and research [1,2]. This paper will present the background related to actigraphy use in studies of persons with Parkinson disease (PD) and present the findings from a pilot study exploring whether limb placement has an influence on sleep actigraphy findings in this population.

Actigraphs are small wristwatch-like accelerometer devices that are placed on limbs to generate scores (based on movement/inactivity in multiple planes) used as indicators of sleep/awake [1,2]. Guidelines recommend the placement of the actigraph monitor on the non-dominant wrist. There is some evidence to suggest that for mobility-challenged children, proximal location at the trunk or shoulder is acceptable when distal location on the non-dominant wrist is not possible [3]. However, it appears that there are no similar studies of persons with PD, and, consequently, evidence-based findings to guide research protocol development regarding the use of actigraphy specific to this population is lacking.

To generate a sleep report, participants wear the actigraph monitor over a number of nights. Data is then downloaded and analyzed to convert frequency and duration of movement in three directional planes to a number of wake/sleep parameters (such as total sleep time, percent of time asleep, total wake time, percent of time awake, number of awakenings) and activity/inactivity [1,2]. There are many commercially available models of actigraphs and algorithms that are selected by the manufacturer are not standardized across all products. Research has found some dissimilarity in regards to the detection and recording of movements [4]. Studies to validate and establish evidence for the use of actigraphy have, for the most part, compared actigraphy to either polysomnography (PSG), or self-report sleep diaries, or both [5,6]. A study by Samson et al. [7] concluded that, with appropriately designated parameters for nap and wake-bout diagnostics, actigraphy could be used to detect segmented sleep. Actigraphy is widely used for identifying sleep patterns that are associated with specific sleep disorders, medical or neurobehavioral disorders, and for testing pharmacological and non-pharmacological interventions [2,8,9]. Reliability and validity studies in healthy adults have demonstrated that actigraphy highly correlates with PSG for differentiating sleep from wake states [10]. Some evidence exists that actigraphy is useful in populations, such as children with attention deficit hyperactivity disorder (ADHD), where PSG might be difficult to obtain [1,2,11].

It appears that actigraphy may have the potential to provide an important and useful tool to study sleep in persons with PD. A study conducted by Wong et al. [12], reported an estimated 0.2% of Canadian adults in private households, and 4.9% of those in residential institutions as having PD. Sleep disorders affect 60% to 90% of persons with PD [13,14,15]. The most common sleep disorders for persons with PD are insomnia, Rapid Eye Movement Behavioral Disorder (RBD), sleep-related breathing disorders, Excessive Daytime Sleepiness (EDS), and Restless Leg Syndrome (RLS) [16,17]. Common causes of sleep disruption for persons with PD are difficulty in changing position during sleep due to bradykinesia, rigidity, and symptoms such as resting tremors during non-rapid eye movement (REM) sleep [18]. Resting tremors may precede micro-awakening, which can then also prevent a return to sleep [15,18]. Anxiety associated with PD and dyskinesia induced by dopaminergic agents can also interfere with sleep and contribute to early morning awakenings and circadian rhythm disruption [15,16,18].

A small number of studies have investigated the usefulness of actigraphy in measuring sleep parameters in persons with PD [19,20,21]. Maglione et al. [9] concluded that actigraphy might be useful for the measurement of total sleep time, sleep efficiency, and wake after sleep onset in persons with mild to moderate PD. Stavitsky et al. [10] showed a correlation of subjective sleep measures with actigraphy-derived estimates of sleep quality, and suggested using actigraphy for assessment of sleep quality in persons with PD. When compared to PSG, actigraphs are more accessible, easier to use, and more cost-effective [9]. Also, when compared to overnight PSG, actigraphy was proposed to be more feasible for use over extended periods of time (i.e., days to weeks), thereby allowing for the collection of information about day-to-day variability in sleep patterns [9,10]. Further, actigraphs can be used in home-based studies, and therefore provide objective information about sleep and wake patterns in the patient’s natural sleep environment [9,10].

However, there are some unknowns and potential drawbacks regarding the use of actigraphy in persons with PD. For example, persons with Parkinson-related dementia may become agitated by wrist monitors and struggle to remove them. Also, actigraphs are sensitive to small movements, and this might make using actigraphy difficult for persons with PD who are experiencing moment-to-moment motor fluctuations (e.g., medication off time), tremors, and dyskinesia [21]. Moreover, the accuracy of actigraphs depends on the sensitivity and specificity of the band-pass filter to yield true activity counts. Since no standardized limit has been set for the band-pass filter, different manufacturers set their own cutoffs [22]. Different cutoffs mean that the generalizability between studies may be compromised. Also, the sensitivity and specificity of a given actigraph model to detect motion will be more accurate in some populations, (i.e., those who best fall within the range of the band-pass filter), and less so in others [21,22]. Therefore, a manufacturer’s default scoring parameters (such as epoch length) may not provide the best data when studying the sleep of persons with PD [22]. Also, as mentioned earlier, the score settings depend on multiple factors, including the sleep measure of interest selected by the investigator or clinician, the device used, and the population assessed [22].

Finally, and the focus of this paper, are issues regarding placement of the actigraph. Available literature shows the variation in scores with actigraph placement on different sites, such as the ankle, hip, and wrist [5,23,24]. Data from studies involving other populations, such as preschoolers, suggests that the wrist, in general, is more accurate for sleep estimation than the placement of the actigraph on other areas of the body [23,24]. One study [23] investigated the agreement between actigraph estimates of physical activity intensity and sedentary behaviour when comparing wrist-worn to hip-worn actigraph accelerometers in preschool children during free-play. They found that total vector magnitude (VM) count output and the mean VM count per minute data collected from the wrist and the hip were significantly correlated (*p* < 0.01). However, in the same study, a notable and systematic difference, with wide limits of agreement, was found between the hip and wrist data [23]. A second study, simultaneously measured sleep using wrist actigraphy, hip actigraphy, and PSG, found that wrist actigraph scored sleep with reasonable specificity, while hip actigraph sleep scores had unacceptably low specificity [24]. To-date, the relationship between placement sites and sleep parameter in persons with PD is inconclusive, and further research is warranted.

In summary, although studies have demonstrated the potential usefulness of actigraphy in measuring sleep parameters in persons with PD [9,10,20,21], there is a lack of clear, evidence-based direction regarding monitor placement [19,23,24]. The data available from studies of other patient populations suggest that the wrist, in general, is more accurate for sleep variable estimation than other placement sites [23,24]. However, no such validating study for persons with PD exists and the unique involuntary motor activity in the limbs of persons with PD, and Parkinson-related dementia and agitation, preclude extrapolation of findings from other patient populations. The purpose of this study is therefore to test the degree of variability of sleep actigraphy scores that are generated during simultaneous placement of monitors on all four limbs (upper/lower, dominant/non-dominant limb).

## 2. Materials and Methods

### 2.1. Study Design

The study applied observational case study design, which is useful to study complex phenomenon within context [25], develop theory, evaluate programs, and to develop interventions [26].

### 2.2. Participants

Following approval from the Research Ethics Board of the University of Alberta, four persons with PD were recruited through a university student-led teaching clinic using a convenience sampling method. The clinic coordinator, not involved in this study, mailed potential participants the information materials and consent form. Inclusion criteria included individuals who: (a) were diagnosed with PD and self-reported having sleep problems; (b) scored > 26/30 (cut point for PD-mild cognitive impairment) on the Montreal Cognitive Assessment (MoCA) [27]; and, (c) were living independently or with a family member in the community.

### 2.3. Procedure and Data Collection Methods

The study was conducted using the Actigraph triaxial sensor (Actigraph WGT 3X-BT, Pensacola, FL, USA) received one actigraph for each of their four limbs. Actigraph monitors were placed into microfleece/velcro closure straps designed for this study to increase comfort and reduce the risk of lower limb constriction during nighttime use. Since persons with PD show motor symptoms, such as tremors and rigidity [16], which might interfere with the actigraph recordings, motor function was assessed with the Movement Disorder Society Unified Parkinson Disease Rating Scale part III (MDS-UPDRS III), which also includes Hoehn and Yahr Staging of Parkinson’s Disease [28]. How to care for and place the actigraph on all four limbs, and how to use the accompanying sleep diary, was demonstrated in the participant’s home. The proprietary Actilife 6 software package (version 20.0, Pensacola, FL, USA, 2017)was set to the Cole-Kripe Sleep Scoring Algorithm option recommended for adult populations and the data collection epochs were set at the longest possible duration (60 s) to minimize as much as a possible the recording of involuntary movements. The algorithm calculated sleep onset latency (SOL), total sleep time (TST), wake after sleep onset (WASO), number of awakening (NA), and overall sleep efficiency (SE) [29,30]. Descriptive analysis was carried out to determine the range and variance of findings comparing all four limbs within each participant.

## 3. Results

Four participants completed the study. Mean age was 72. Five years, all were right-hand dominant males (Table 1). The comparative data across all four limbs are detailed in Table 2. The spread in sleep actigraphy scores for SE, TST, NA and WASO, recorded in the dominant arm were similar to the scores in non-dominant arm (Table 3). As found in the upper limbs, the spread in sleep variable scores comparing left and right lower limbs was also similar (Table 3). However, on average, for each participant, the WASO scores were higher in the right lower limb than left lower limb (Table 3). When comparing each participant’s right upper limb, to his right lower limb, revealed that the right upper limb score for sleep efficiency was higher (Table 3). Conversely, the right lower limb NA scores were lower than the right upper limb NA scores (Table 3). Notably, WASO scores for the right lower limb were markedly less than the WASO scores for the right upper limb. For all four participants, the SE and TST scores were less from the lower limb monitors as compared to the upper limb monitors. Upper limb monitors generated slightly higher NA and WASO scores when compared to lower limbs. As illustrated in Table 3, TST and WASO were the least stable readings with the widest variance in scores across the four limbs.

## 4. Discussion

To the best of our knowledge, this is the first study to examine the consistency of sleep actigraphy scores in persons with PD when measured across all four limbs simultaneously. Similar to Maglione et al. [9], analysis found that there were minimal differences in the measurement of the sleep variables between the dominant and non-dominant arm. However, there was a notable discrepancy between certain lower and upper limb scores. The SE and TST scores were higher in lower limbs when compared to upper limbs. Given that persons with PD experience upper extremity tremor and other motor problems (such as lower limb rigidity), which might interfere the actigraph readings [28,31], our findings seem to support that there is an overestimation of sleep quality for actigraphs placed on lower limbs. These results align with previous findings that actigraphs placed on healthy young adults at hip level tend to overestimate sleep [24]. Conversely, it is possible that actigraphy placement on upper limbs leads to underestimation of sleep and larger studies, with polysomnography, will be required to determine the nuances of this question. Number of awakenings (NA) and WASO scores were markedly decreased in lower limbs when compared to upper limbs, and we speculated that these findings might be due to poor detection of the awake state when the monitor is placed on the ankle of someone with a Parkinson-related motor impairment. Previous studies of healthy adults reported that monitor placement at hip level yields lower NA and WASO scores due to poor sleep detection [24]. Coupled with the PD motor complications mentioned earlier, this suggests that actigraphy placement at the hip or ankle is not reliable for this population. Our pilot results determined that, overall, considerable variability exists in simultaneously collected sleep indicator scores within an individual when actigraphs are placed on different sites. These results are similar to previous studies of preschoolers and high-altitude travelers [5,32]. Other research also suggested the non-dominant wrist as the most suitable site for placement of actigraphs [19]. However, for persons with PD, issues of dementia and agitation, tremor, and rigidity, are all considerations that can complicate the selection of the actigraph placement site. The lack of evidence-based guidelines for actigraphy use specific to the Parkinson population is an important gap in the research and further studies, with polysomnography and video recording comparators, are required to refine our understanding of the issues that are related to selecting actigraph placement sites for persons with PD.

Although there has been an increasing use of actigraphy to quantitatively record the symptoms of PD (such as activity and sleep disturbance), no studies so far have undertaken an evaluation to guide the placement of actigraphs in research protocols. Thus, this study contributes important preliminary data to deepen our understanding of the use of sleep actigraphy in studies involving persons with PD. A specific recommendation that can be made at this point for studies involving intrapersonal comparisons is to maintain the same placement site across all of the nights, and if relocation of the monitor is required because of discomfort or agitation, then the data collection should be restarted.

Limitations of the study include the small sample size and the lack of generalizability. Our sample excluded persons with greater than mild dementia and the influence of this remains to be studied. One participant reported difficulties applying the actigraphs. Although no one else reported problems, the possibility of compromised data because of problems applying and wearing the actigraph monitors exists. Furthermore, due to reduced cognitive abilities in some persons with PD, it is possible that the requirement of a self -report sleep dairy to collect time of donning and removing the actigraph may have introduced some error. Lack of a PSG comparator and confirmed sleep disorder diagnosis were also limitations and findings may have been biased by undiagnosed comorbid sleep problems (e.g., breathing disorder leading to arousals after sleep apneas and REM sleep behavior disorders), and by limb movements, whilst asleep that could be misinterpreted by the software as awakenings. Lacking a PSG benchmark, data recorded by the actigraphs could not be compared to sleep indicators that were not motion-dependent. Future efforts are needed to build on this preliminary study and guide suitable placement site for sleep actigraphy use in research involving persons with PD.

## 5. Conclusions

Over the last two decades, actigraphy has become an accessible and widely used assessment tool in sleep research and clinical practice. However, knowledge regarding the implementation of actigraphy in studies of persons with PD is sparse. This study contributes preliminary data regarding suitable actigraph placement site depending on the sleep indicator(s) of interest. This study also suggests that placement of the actigraph on either the dominant or non-dominant wrist may not affect certain sleep scores to the extent that placement on upper versus lower limbs would have. Furthermore, the possibility of over and underestimation of sleep scores dependent on limb placement will require closer study with simultaneous PSG. We are only able to speculate at this time and further research is much needed in this area.

## Figures and Tables

**Table 1:** Demographics.

	Gender	Age (Years)	Right Hand Dominance	MoCA	UPDRS Part III Aggregate Score	H &Y Stage
Participant 1	M	54	Yes	29	35	2
Participant 2	M	83	Yes	27	72	3
Participant 3	M	71	Yes	28	52	2
Participant 4	M	79	Yes	27	58	2
Mean		72.5		27.75	54.25	2

Key: MoCA = Montreal cognitive assessment; MDS-UPDRS = Movement Disorders Society Sponsored Unified Parkinson’s Disease Rating Scale; H & Y Stage = Hoehn-Yahr Stage.

**Table 2:** Actigraph readings across participants with simultaneous limb placement (rounded off to nearest whole number).

	Participant 1	Participant 2	Participant 3	Participant 4
Right lower limb				
SE (%)	65–83	98–100	88–96	87–95
TST (min)	283–344	427–448	369–503	369–495
WASO (min)	62–152	2–6	13–50	20–56
NA	10–15	1–2	3–11	4–13
Avg. A (min)	6–10	2–6	4–12	2–5
Right upper limb				
SE (%)	47–74	97–100	58–87	79–94
TST (min)	206–297	425–441	247–444	336–469
WASO (min)	95–229	1–7	52–181	35–89
NA	9–14	1–3	12–28	4–17
Avg. A (min)	9–16	1–4	3–6	3–6
Left lower limb				
SE (%)	76–84	98–100	82–94	91–96
TST (min)	278–364	427–448	345–490	387–494
WASO (min)	71–108	1–6	24–72	19–38
NA	9–11	1–2	5–24	5–12
Avg. A (min)	7–14	1–6	3–5	3–5
Left upper limb				
SE (%)	46–67	98–100	59–83	87–94
TST (min)	202–298	427–448	246–443	380–461
WASO (min)	134–233	1–7	70–167	20–57
NA	8–13	1–4	12–34	3–15
Avg. A (min)	10–18	1–4	4–7	3–9

Key: SE = sleep efficiency; TST = total sleep time; WASO = wake after sleep onset; NA = number of awakening; Avg. A = average awakening.

**Table 3:** Within-participant variance of actigraph scores.

	Participant 1 Range	Participant 2 Range	Participant 3 Range	Participant 4 Range
Lower limbs				
SE (%)	65.06–83.78	98.62–99.54	82.14–96.14	86.82–95.71
TST (min)	278–364	427–448	345–503	369–495
WASO (min)	62–152	1–6	13–72	19–56
NA	9–15	1–4	3–24	4–13
Avg. A(min)	6–14	1–6	3–12	3–5
Upper limbs				
SE (%)	46.44–73.61	96.92–99.77	57.58–87.38	79.06–94.13
TST (min)	202–298	416–448	246–444	336–469
WASO (min)	95–233	1–7	52–181	20–89
NA	8–14	1–4	12–34	3–17
Avg. A (min)	9–18	1–4	3–7	3–9

Key: SE = sleep efficiency; TST = total sleep time; WASO = wake after sleep onset; NA = number of awakenings; Avg. A = average awakening.
